# Inflammatory bowel disease and the heart: how trained immunity shapes cardiovascular outcomes

**DOI:** 10.3389/fimmu.2026.1935682

**Published:** 2026-10-19

**Authors:** Shuanghua Liang, Long Han

**Affiliations:** Organization Hunan Aerospace Hospital, The Affiliated Aerospace Hospital of Hunan Normal University, Changsha, China

**Keywords:** atherosclerosis, cardiovascular disease, epigenetics, inflammatory bowel disease, innate immune memory, trained immunity

## Abstract

Inflammatory bowel disease (IBD), comprising Crohn’s disease and ulcerative colitis, is a chronic relapsing intestinal inflammatory disorder. Cardiovascular disease (CVD) is a leading cause of death in patients with IBD, with elevated risks of myocardial infarction, heart failure, stroke, and venous thromboembolism that are independent of traditional risk factors. Disease activity and histological inflammation independently predict major adverse cardiovascular events, and Crohn’s disease and ulcerative colitis differ in cardiovascular risk profiles. This residual risk points to immune mechanisms that remain unclear. Trained immunity (TI), an innate immune memory induced by epigenetic and metabolic reprogramming, may link chronic intestinal inflammation to cardiovascular injury. This review integrates epidemiological and mechanistic evidence, outlines cardiovascular risk features in IBD, examines the molecular basis of TI and its roles in atherosclerosis, myocardial injury, and heart failure, and evaluates candidate biomarkers and targeted therapeutic strategies. We also discuss current limitations and challenges in clinical translation and propose future research directions, to inform mechanistic studies and clinical interventions for IBD-associated CVD.

## 1 Introduction

IBD, comprising Crohn’s disease and ulcerative colitis, affects over 7 million people worldwide (1). CVD has emerged as a leading cause of morbidity and mortality in IBD patients (2). A recent meta-analysis of 2.2 million individuals confirmed increased risks of myocardial infarction, heart failure, stroke, and venous thromboembolism (VTE) in IBD (3), and a Swedish nationwide cohort showed that heart failure risk remains elevated for at least 20 years after diagnosis (4). These findings position CVD as a clinically actionable complication of IBD, requiring early risk stratification and integrated management (2).

Traditional cardiovascular risk factors are prevalent in IBD and contribute to this risk (5). However, even after adjustment, patients with IBD exhibit independent residual cardiovascular risk (6), and adolescent males with Crohn’s disease (CD) show increased inherited hypercoagulability despite normal conventional risk profiles (7). Mendelian randomization supports causal links between atherosclerosis and IBD (8) and between ischemic stroke and both CD and ulcerative colitis (9). These observations challenge the predominant role of conventional factors and point to more complex immune mechanisms.

TI provides a novel framework for this independent risk. TI refers to innate immune cells undergoing epigenetic and metabolic reprogramming after initial exposure to microbial or endogenous danger signals, acquiring memory-like properties and mounting enhanced inflammatory responses to subsequent heterologous stimuli (10). In IBD, gut dysbiosis and barrier dysfunction facilitate microbial translocation, priming circulating monocytes into a trained state (11). After migrating to the vascular wall, these “trained” monocytes show exaggerated responses to atherosclerosis-related stimuli, accelerating plaque formation and cardiovascular events (12). This hypothesis offers a new perspective on the immunological connection between IBD and CVD.

Focusing on this hypothesis, this review summarizes the epidemiological evidence for increased cardiovascular risk in IBD patients, analyzes the mechanisms that induce TI, elaborates its specific role in promoting endothelial dysfunction, atherosclerosis, and thrombosis, and discusses therapeutic strategies targeting TI. Our aim is to provide a new theoretical basis and clinical guidance for cardiovascular risk management in IBD patients (13).

## 2 Epidemiological evidence linking IBD to cardiovascular risk

IBD increases cardiovascular risk, particularly in younger patients and during active disease (3, 14). Active IBD raises the risk of major adverse cardiovascular events (MACE) by ~50% versus remission, and histological inflammation independently predicts MACE, remaining associated with MI and heart failure even during clinical remission (15). In Chinese patients with IBD, ischemic heart disease (IHD) risk is markedly increased and peaks in young women (16); in Japanese patients with UC, cumulative MACE incidence rises with age, with diabetes and atrial fibrillation as major contributors (17). CD and UC differ in risk profile: CD carries higher IHD risk, whereas UC is more strongly associated with venous thromboembolism (VTE) (14, 18, 19). NOD2, the principal CD susceptibility gene, aggravates myocardial ischemia/reperfusion injury through JNK/p38 MAPK/NF-κB signaling (20), and NOD2 up-regulation has been detected in coronary atheroma lesions (21). In UC, barrier dysfunction permits LPS translocation into the systemic circulation, where LPS binding to TLR4 on monocytes induces tissue factor expression and initiates the extrinsic coagulation cascade (22, 23). A Swedish nationwide cohort of 55,252 patients confirmed a doubled VTE risk in IBD (24). Thus, CD-predominant NOD2 signaling and UC-predominant TLR4–tissue factor activation represent distinct immunological routes converging on subtype-specific cardiovascular phenotypes.

Mendelian randomization supports causal links between CD/UC and chronic heart failure, but not atrial fibrillation or coronary heart disease (25); shared genetic loci with IHD and atrial fibrillation further explain these subtype-specific risks (26). Flares, particularly around hospitalization (27), and elevated C-reactive protein (CRP) or fibrinogen (16, 28) increase risk, supporting deep remission as a preventive strategy.

Conventional risk factors show a distinct pattern. Dyslipidemia manifests as the “lipid paradox”, low total cholesterol and low-density lipoprotein (LDL), impaired high-density lipoprotein (HDL) function, and elevated triglycerides (12), with inflammation-driven reprogramming that may render LDL smaller, denser, and more atherogenic (29). The prevalence of hypertension and diabetes is comparable to that in the general population, but inflammation exacerbates vascular damage (16, 30); Mendelian randomization supports UC–heart failure causality independent of hypertension or coronary disease (25). Smoking has opposing effects in UC and CD (31), so risk stratification should integrate subtype, disease activity, inflammatory markers, and medication effects (13).

Even after adjustment for traditional factors, IBD confers independent residual cardiovascular risk. CRP and interleukin-6 (IL-6) correlate with risk independently of lipids, and bowel resection reduces CRP while improving coronary function (32). Despite low LDL levels, pro-inflammatory cytokines promote endothelial dysfunction, platelet aggregation, and coagulation (33, 34), and lipid quality predicts coronary function better than lipid levels (35). Gut dysbiosis and barrier dysfunction contribute to a “gut–heart axis” (36): endotoxin translocation triggers systemic inflammation and endothelial dysfunction (37, 38), microbial metabolites such as trimethylamine N-oxide (TMAO) promote foam cell formation and plaque instability (39), and changes in bile acid and short-chain fatty acid (SCFA) metabolism modulate lipid metabolism and inflammation (40). Microbiota-targeted interventions may therefore reduce cardiovascular risk in IBD (38).

## 3 Molecular mechanisms and regulatory networks of TI

### 3.1 Definition and conceptual framework

TI is an innate immune memory induced by epigenetic and metabolic reprogramming, enabling monocytes/macrophages to mount enhanced non-specific responses to secondary stimuli (10). Unlike adaptive immunity, TI does not rely on RAG-mediated recombination but on persistent histone modifications, DNA methylation, and metabolic shifts (10). Critically, bone marrow hematopoietic stem cells (HSCs) can also acquire this memory, sustaining myeloid output and systemic inflammation long after the initial trigger (41). In IBD, chronic gut inflammation may prime circulating monocytes into a pre-activated state that overreacts to atherogenic stimuli, thereby accelerating CVD (42).

### 3.2 Epigenetic and metabolic reprogramming

At the epigenetic level, H3K4me3 and H3K27ac enrichment at pro-inflammatory promoters keeps TNF and IL6 poised for rapid transcription (43), whereas promoter hypomethylation and increased chromatin accessibility establish a stable memory that renders cells hyper-responsive to cardiovascular risk factors (44). Metabolically, trained cells shift from oxidative phosphorylation to aerobic glycolysis, generating ATP and biosynthetic precursors (45). Glutaminolysis and the mevalonate pathway supply α-ketoglutarate and acetyl-CoA for histone demethylation and acetylation, reinforcing pro-inflammatory gene expression (46). Mitochondrial remodeling further increases reactive oxygen species, which activate the NLRP3 inflammasome and stabilize HIF-1α, creating a positive feedback loop linked to endothelial dysfunction and myocardial injury (45, 47).

### 3.3 Induction and resolution in IBD

TI is induced by exogenous microbial products (β-glucan, BCG) and endogenous danger signals (oxLDL, MSU) (44, 45, 47). In IBD, barrier dysfunction allows microbial translocation and raises circulating oxLDL, chronically priming innate immune cells and amplifying vascular inflammation (44). TI resolution requires reversal of epigenetic marks and restoration of metabolic homeostasis; however, recurrent flares can drive TI exhaustion or immune paralysis, characterized by reduced responsiveness and increased IL-10 (48). Understanding this dynamic balance is essential for targeting cardiovascular complications in IBD.

## 4 Induction and characteristics of TI in IBD

### 4.1 Gut dysbiosis as a trigger of TI

The dysbiotic signature of IBD is consistently characterized by a loss of short-chain fatty acid (SCFA)-producing commensals (Faecalibacterium prausnitzii, Roseburia, Coprococcus) and an expansion of adherent-invasive Escherichia coli (AIEC) and Escherichia-Shigella (49–51). This ecological shift is not merely associative: it directly impairs barrier integrity, allowing bacterial cell wall components (LPS, peptidoglycan) to engage pattern recognition receptors on monocytes/macrophages and thereby provide the initial trigger for TI (11).

Changes in microbial metabolites play a more refined role in epigenetic reprogramming. Metabolite profiling consistently shows depleted SCFA (butyrate, acetate, propionate) levels in IBD (52–54). Mechanistically, SCFAs normally restrain pro-inflammatory gene expression by inhibiting HDACs; their depletion therefore removes this brake and promotes M1 macrophage polarization (55). In parallel, secondary bile acids (lithocholic acid, deoxycholic acid) with anti-inflammatory activity decline, whereas primary bile acids relatively increase (50, 51), exacerbating inflammation and metabolic disturbance via FXR/TGR5 signaling (56, 57). Together, these shifts create a pro-inflammatory microenvironment for innate immune cells, driving persistent reprogramming through histone modification and DNA methylation. Thus, global alterations in metabolites provide essential “fuel” for the establishment of TI.

Intestinal barrier dysfunction bridges local dysbiosis and systemic TI. In IBD, the expression of tight junction proteins (zonula occludens-1, occludin) decreases and the mucus layer thins, producing a “leaky gut” (11). Microbial products (LPS, peptidoglycan, bacterial DNA) translocate into the systemic circulation, continuously stimulating Kupffer cells and monocytes and keeping them in a low-grade activated state (11). This persistent stimulation further induces reprogramming of hematopoietic stem/progenitor cells (HSPCs) in the bone marrow, generating more pro-inflammatory monocytes and establishing a systemic TI state that exacerbates extra-intestinal complications, including CVD (58). Consequently, through trigger signals, metabolic changes, and barrier translocation, gut dysbiosis acts as the initiating link between local intestinal inflammation and the induction of systemic TI. This process provides the biological foundation for understanding how TI influences cardiovascular outcomes, a topic explored in subsequent chapters.

### 4.2 TI phenotype of circulating monocytes in IBD patients

Circulating monocytes from patients with IBD show a typical TI phenotype, with enhanced pro-inflammatory responses to heterologous stimuli. Upon secondary stimulation with LPS or Escherichia coli, their capacity to produce TNF-α, IL-1β, and IL-6 is significantly greater than that of healthy controls (59). In patients with CD, classical monocytes (CD14++CD16-) show an “exhausted” phenotype (increased CD38, decreased IL-1β) after LPS stimulation, suggesting functional alterations under sustained inflammatory pressure (60). Monocytosis serves as a biomarker of disease severity in IBD and correlates with hospitalization rates, surgery rates, and emergency department visits (61).

Monocyte subset distribution also changes markedly: the proportion of classical monocytes increases, and the pro-inflammatory function of intermediate monocytes (CD14++CD16+) is enhanced. In UC patients with anxiety or depression, the proportions of CD14++CD16+ and CD14+CD16++ monocytes are elevated, and their phagocytic capacity is impaired (62). CD30L expression on CD14++CD16+ cells is increased and correlates positively with disease severity (63). Subset phenotypes can distinguish CD from UC and reflect mucosal inflammatory status (64), and biologic therapy can alter their distribution (e.g., CD44 expression) (65).

Consistent with the epigenetic programs defined in Section 2.2, monocytes from IBD patients show elevated H3K4me3 and H3K27ac at pro-inflammatory gene promoters (66), and these marks correlate with endoscopic and clinical activity (48). The mechanistic basis of these marks is described in Section 2.2. Targeted delivery of HDAC inhibitors to CES1+ monocytes improves IBD in mouse models (67), confirming the functional relevance of histone acetylation in this disease context.

### 4.3 Reprogramming of bone marrow hematopoietic stem/progenitor cells

IBD-associated systemic inflammation affects bone marrow HSPCs, inducing epigenetic reprogramming that skews differentiation toward the myeloid lineage and produces more pro-inflammatory monocytes. Two distinct lines of evidence converge on the same conclusion: HSPC homeostasis is disrupted in IBD, leading to excessive proliferation of Ly6C+ monocytes (68), and gut leakage in a UC mouse model increases serum LPS and IL-1β, up-regulating myeloid differentiation pathways (69). Single-cell RNA sequencing further reveals increased lactylation modification in HSPCs from UC patients, pointing to metabolic-epigenetic regulation (70), while circulating TNF-α can affect the bone marrow microenvironment and drive myeloid skewing (71).

TI memory in HSPCs can persist for months, maintaining monocyte activation even after intestinal inflammation subsides. In severe COVID-19 patients, overgrowth of gut Candida albicans induces transcriptional reprogramming of HSPCs, causing long-lasting (up to one year) changes in granulocyte-monocyte progenitors (GMPs) (72). After resolution of inflammation in a UC mouse model, HSPCs retain their myeloid-biased differentiation and show an enhanced inflammatory response upon re-challenge with periodontitis (69). While mechanistically informative, these findings come from non-IBD contexts (COVID-19) or a single IBD mouse model and should not be directly equated with human IBD HSPC memory.

Bone marrow transplantation experiments further show transfer of HSPC memory. Transplanting bone marrow from colitis mice into healthy recipients induces a similar myeloid bias in the recipient’s HSPCs, with elevated circulating monocytes and pro-inflammatory cytokines (71). Blocking TNF-α signaling partially reverses this effect, indicating that TNF-α is a key mediator (71). These findings provide the strongest available causal evidence linking colitis-associated inflammation to persistent HSPC reprogramming in animal models. However, whether such transferable HSPC memory occurs in human IBD and contributes to cardiovascular risk remains to be demonstrated. Thus, from gut dysbiosis to phenotypic changes in circulating monocytes to persistent reprogramming of bone marrow HSPCs, TI establishes a cascade from local to systemic inflammation in preclinical models, with human translation still pending.

## 5 TI in atherosclerosis: a concise, IBD-relevant mechanistic framework

As TI in atherosclerosis has been extensively reviewed, this section is restricted to mechanisms that are necessary for interpreting IBD-specific amplification or that are directly modifiable in IBD. We do not attempt a comprehensive overview of atherogenesis. As detailed in Section 2, TI confers a pro-inflammatory memory on monocytes/macrophages and HSPCs (45, 47). Rather than restating these mechanisms, this section focuses on how they are chronically primed and repeatedly restimulated in IBD. In atherosclerosis, these programs converge on a common effector cascade: CCR2/CX3CR1-dependent monocyte recruitment, MMP-mediated matrix degradation, cytokine-driven endothelial activation, foam-cell formation, neovascularization, and tissue-factor-dependent hypercoagulability, collectively promoting plaque vulnerability and rupture (31, 37, 44, 73, 74). The key point for IBD is not merely the presence of these pathways, but their chronic priming by three IBD-specific amplifiers that may intensify TI beyond general induction. These include sustained LPS translocation across a compromised intestinal barrier, which provides continuous TLR4 stimulation (11); a dysbiotic metabolome depleted in tolerogenic short-chain fatty acids and enriched in primary bile acids, which removes HDAC-mediated restraint on pro-inflammatory genes and reinforces innate immune reprogramming (50, 51, 55); and NOD2, the principal Crohn’s disease susceptibility gene, which is required for microbial sensing and TI induction, yet its variants impair bacterial clearance and may dysregulate the balance between TI and immune tolerance (48). Such amplifiers may distinguish IBD-associated TI from TI in other chronic inflammatory conditions and help explain why IBD lowers the threshold for TI induction and sustains TI after intestinal inflammation appears controlled (75). These mechanistic inferences should be regarded as hypothesis-generating rather than established causal pathways in IBD (**Table 1**; **Figure 1**).

**Table 1:** TI mechanisms in IBD-associated CVD.

Mechanism	Key players	Cardiovascular consequences in IBD	References
Epigenetic reprogramming	H3K4me3, H3K27ac, DNA hypomethylation, open chromatin	Persistent pro-inflammatory state of monocytes/macrophages; accelerated atherosclerosis	(12, 30, 31, 44, 45)
Metabolic remodeling	Aerobic glycolysis, glutaminolysis, mevalonate pathway, mitochondrial ROS	Supplies substrates for epigenetic changes; promotes endothelial dysfunction and myocardial injury	(45–47)
Key inducers	Microbial products (LPS, β-glucan), oxLDL	Repeated innate immune training via gut barrier dysfunction and dysbiosis	(31, 44, 45, 47)
HSC reprogramming	TNF-α-driven myeloid bias, long-lasting epigenetic memory	Sustained production of trained monocytes, even after intestinal remission	(69, 71, 72)
Atherosclerosis effects	CCR2/CX3CR1↑, MMPs↑, adhesion molecules↑, foam cells↑, neovascularization	Plaque vulnerability, rupture, thrombosis	(31, 44, 73)
Cardiac injury	Acute: ischemia/reperfusion injury(IRI) exacerbation, cardiomyocyte death	Worse post-MI outcomes; Heart failure with preserved ejection fraction (HFpEF); coronary microvascular dysfunction	(4, 32, 76, 77)
	Chronic: fibroblast activation, fibrosis, diastolic dysfunction		

**Figure 1:**
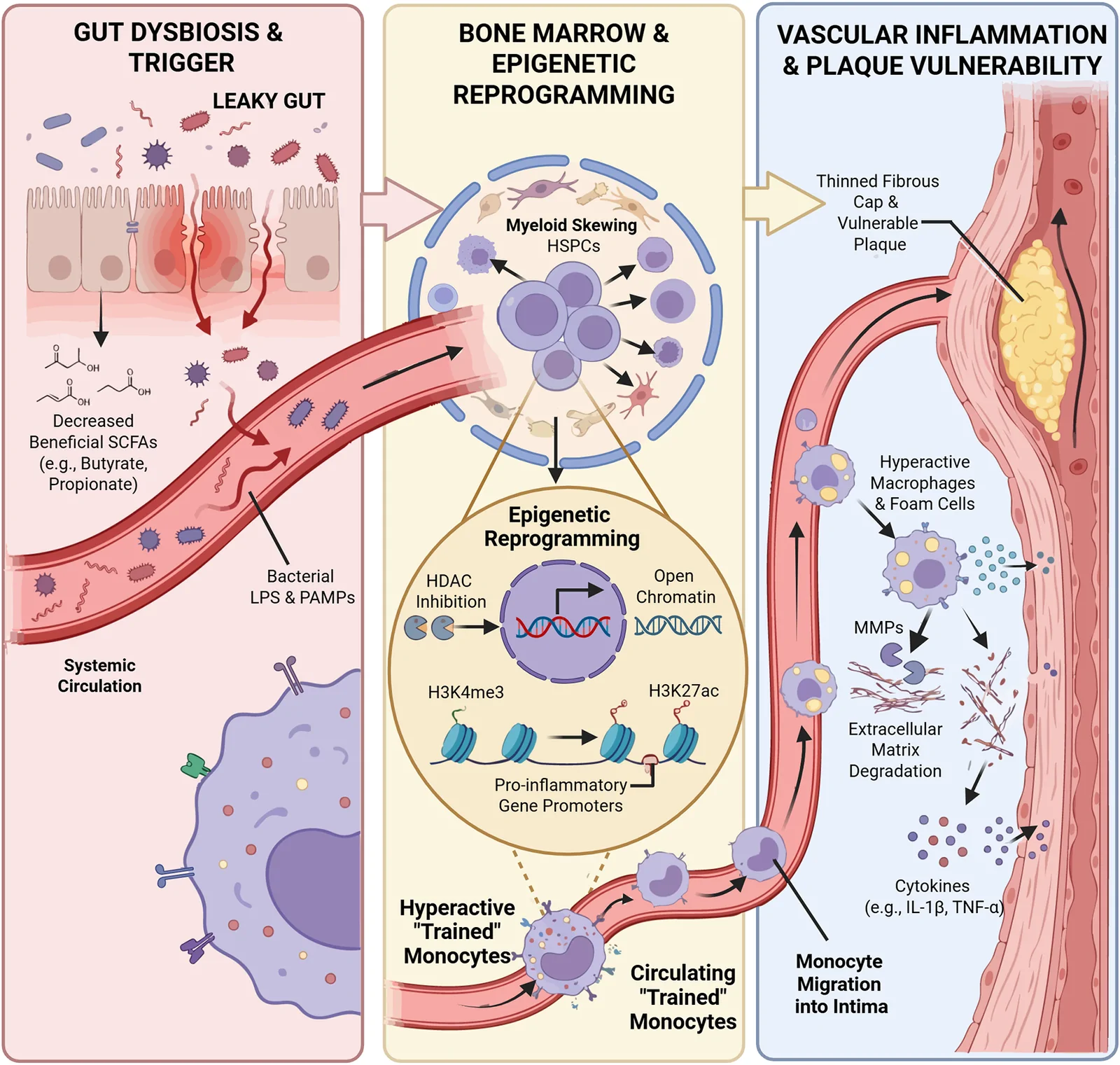
Overview of the mechanisms by which TI mediates cardiovascular injury in IBD. Created in biorender.

Gut dysbiosis and barrier dysfunction promote microbial translocation. Microbial products and oxidized LDL prime monocytes and hematopoietic stem/progenitor cells through pattern recognition receptors. Epigenetic reprogramming and metabolic remodeling establish a persistent pro-inflammatory memory. Trained monocytes migrate to the vascular wall, amplifying monocyte recruitment, matrix degradation, endothelial activation, foam cell formation, and hypercoagulability. In the heart, trained macrophages release pro-inflammatory cytokines, inducing cardiomyocyte apoptosis, fibrosis, and diastolic dysfunction. Solid arrows indicate established mechanistic links; dashed arrows indicate inferred links.

## 6 Amplified effects and specific cardiovascular outcomes of TI in the context of IBD

The preceding section outlined the core mechanisms by which TI contributes to general atherosclerosis. However, these general pathways do not fully capture the IBD-specific amplification that shapes cardiovascular outcomes. As a chronic, relapsing disease, IBD involves a persistent inflammatory burden and translocation of microbial products, which can pathologically amplify the aforementioned TI pathways and produce several disease-specific manifestations. To integrate these outcomes into the mechanistic narrative from the outset, this chapter analyzes how IBD-associated TI accelerates atherosclerotic progression, and further distinguishes its differential impact on acute myocardial injury (focusing on ischemia/reperfusion and cardiomyocyte death) versus chronic heart failure (focusing on myocardial fibrosis and diastolic dysfunction), with particular emphasis on how IBD as an underlying disease modifies cardiovascular outcomes. The evidence supporting each level of this framework is graded in **Table 2**.

**Table 2:** Evidence grading for TI-mediated cardiovascular injury in IBD.

Evidence level	Key findings	Applicability to IBD
Human IBD cohorts	TI-like monocyte phenotype; TI markers associated with CV risk	Associative; causality not established
IBD animal models	Colitis-induced HSPC reprogramming; transferable myeloid bias	Causal in models; human translation pending
Non-IBD inflammatory models	HSPC reprogramming in COVID-19; trained hypercoagulability	Mechanistic parallels; not IBD-specific
Hypothetical mechanism	TI may link gut dysbiosis to atherosclerosis, myocardial injury, and HF	Hypothesis-generating; requires human validation

Supporting references are cited in the corresponding sections of the main text. The final row synthesizes evidence discussed in Sections 3–5.

### 6.1 TI accelerates atherosclerosis progression in IBD patients

Clinical imaging studies show that patients with IBD have increased carotid intima-media thickness and higher coronary artery calcium scores. These changes correlate positively with monocytic TI markers, suggesting that systemic inflammation in IBD promotes early vascular wall injury through TI (78–80). Mechanistically, chronic inflammation in IBD keeps monocytes/macrophages in a persistently “pre-activated” state. This state then exaggerates the processes described in Section 4, namely monocyte chemotaxis into the vessel wall, excessive secretion of MMPs, and up-regulation of endothelial adhesion molecules followed by an inflammatory cascade (31, 81). Notably, the already high serum MMP levels in patients with IBD (82) combine with the further MMP up-regulation induced by TI, creating an additive effect that markedly increases the risk of plaque rupture. Animal models provide causal evidence: transplanting “trained” monocytes from IBD mice into healthy recipients accelerates atherosclerotic plaque formation in non-IBD settings (83). However, direct evidence from IBD mouse models is currently lacking, and this extrapolation should be regarded as inferential.

### 6.2 TI and myocardial injury in IBD patients

Patients with IBD have worse outcomes after acute MI. As expected from the TI phenotype described in Sections 2 and 3, chronic inflammation keeps innate immune cells in a pre-activated state; during acute MI, trained macrophages produce excess pro-inflammatory cytokines and ROS, enlarging infarct size and impairing functional recovery (74). This mechanistic inference is based on TI studies in non-IBD contexts; the specific contribution of IBD-associated TI to worsened post-MI outcomes in patients with IBD has not been directly demonstrated.

Trained macrophages infiltrate the infarct zone and release large amounts of TNF-α, IL-1β, and IL-6, inducing cardiomyocyte apoptosis and necrosis and promoting fibrotic scar formation. Coronary flow velocity reserve studies show that IBD patients have microvascular dysfunction, which improves after surgical resection of diseased bowel and correlates with a decrease in inflammatory markers (32). In active IBD, serum high-sensitivity troponin levels are elevated and correlate positively with C-reactive protein and IL-6; case reports also indicate that an acute flare of ulcerative colitis can trigger acute coronary syndrome (84). Thus, monitoring TI status may help predict and prevent myocardial injury in patients with IBD.

### 6.3 TI and the risk of heart failure in IBD patients

Unlike acute myocardial injury, the elevated risk of chronic HF in patients with IBD arises mainly from TI-induced myocardial fibrosis and diastolic dysfunction. Large cohort studies show that patients with biopsy-confirmed IBD have a significantly increased risk of HF during long-term follow-up, which persists for more than twenty years across all subtypes (4); meta-analyses confirm an increased event rate (85); Mendelian randomization supports a causal relationship (25). Heart failure with preserved ejection fraction (HFpEF) is particularly prominent and closely linked to trained-immunity-associated microvascular inflammation and myocardial fibrosis (86). This association is currently supported by preclinical and translational evidence rather than direct human IBD studies establishing TI as the causal mediator.

Trained myeloid cells in the heart release TGF-β and IL-1β, activating cardiac fibroblasts and increasing extracellular matrix deposition, thereby reducing myocardial compliance. This state is sustained by epigenetic reprogramming in bone marrow-derived monocytes and macrophages, marked by H3K4me3 enrichment at pro-inflammatory gene promoters, which maintains hyperresponsiveness. Coronary microvascular dysfunction, reflected by reduced coronary flow velocity reserve in IBD patients, further aggravates diastolic dysfunction and improves after resection of diseased bowel (32). Coronary microvascular dysfunction is also present: coronary flow velocity reserve is significantly reduced in IBD patients, with IBD itself and inflammatory markers being independent predictors; it improves after surgical resection of the diseased bowel (32). Chronic myocardial ischemia and interstitial fibrosis caused by microvascular damage further aggravate diastolic dysfunction.

In summary, IBD-associated TI affects the heart through two relatively distinct pathways: acute myocardial injury, mainly by worsening ischemia/reperfusion injury and cardiomyocyte death; and chronic heart failure, mainly by activating fibroblasts, promoting myocardial fibrosis, and impairing diastolic function. Both share microvascular dysfunction as a common background, but their core pathological mechanisms and clinical outcomes differ, requiring differentiated intervention strategies.

## 7 TI as a biomarker for IBD-associated CVD

### 7.1 Identification of circulating TI markers

TI markers in patients with IBD can be assessed at three levels: functional, epigenetic, and metabolic. Functionally, the capacity of peripheral blood monocytes to produce cytokines after stimulation serves as a surrogate marker. Monocytes from IBD patients produce more TNF-α and IL-6 upon lipopolysaccharide stimulation, reflecting functional reprogramming. However, this assay is complex and difficult to standardize. Epigenetically, histone modifications (e.g., H3K27 trimethylation) at the promoters of specific genes are more stable and specific (43), but chromatin immunoprecipitation sequencing remains costly. Metabolically, serum lactate and the glutamine/glutamate ratio can indirectly reflect metabolic reprogramming; α-ketoglutarate, as a cofactor for histone demethylases, influences epigenetic modifications (46) and is readily measurable in routine laboratory tests. In summary, a multi-dimensional combination of these markers may help accurately identify the TI state and provide new tools for cardiovascular risk prediction.

### 7.2 Association between TI markers and cardiovascular risk

The capacity of monocytes from patients with IBD to produce TNF-α independently correlates with carotid plaque burden and future cardiovascular events, and its predictive value outperforms C-reactive protein (13, 32). Mechanistically, chronic inflammation in IBD drives epigenetic reprogramming of bone marrow hematopoietic stem cells, endowing monocytes/macrophages with a TI phenotype and shifting their metabolism toward aerobic glycolysis, which fuels the synthesis of pro-inflammatory cytokines (87, 88). Thus, TNF-α-producing capacity directly reflects the pro-atherogenic immune state.

Among epigenetic markers, H3K4me3 levels at the TNF promoter are of particular interest. The mechanistic basis of this epigenetic mark is described in Section 2.2. In IBD patients, even during clinical remission, persistently high H3K4me3 levels at the TNF locus in bone marrow progenitor cells endow monocytes with the potential to rapidly produce TNF-α (89). Therefore, H3K4me3 measurement captures the latent pro-inflammatory potential of the body and provides more stable risk stratification information than static inflammatory markers.

Elevated serum lactate has been reported in patients with IBD as a marker of metabolic reprogramming, and IBD is associated with increased cardiovascular morbidity (90). As introduced in Section 2.3, TI is characterized by enhanced glycolysis and abundant lactate production. In IBD, circulating monocytes/macrophages exhibit hyperactive glycolysis, leading to a systemic increase in serum lactate (91). Lactate acts as a signaling molecule, activating receptors or serving as a substrate for lactylation, thereby amplifying inflammation (70, 90). Serum lactate levels directly reflect the degree of TI activation, and targeting this metabolic pathway holds therapeutic potential (92) (**Table 3**).

**Table 3:** Biomarkers for TI in IBD.

Category	Marker	Rationale	Evidence in IBD	References
Functional	Monocyte TNF-α after ex vivo LPS stimulation	Reflects TI status; predicts carotid plaque burden and CV events	Independent of CRP; outperforms traditional markers	(13, 32)
Epigenetic	H3K4me3 at TNF promoter	Captures latent pro-inflammatory potential	Predicts CV events; elevated in IBD progenitors	(89, 93)
Metabolic	Serum lactate, α-ketoglutarate	Indicates glycolysis/glutaminolysis activity	Lactate associated with cardiovascular morbidity in IBD	(46, 90, 92)

### 7.3 Clinical translation of TI biomarkers: feasibility, limitations, and IBD-specific priorities

Current candidate markers—monocyte TNF-α production after ex vivo LPS stimulation … should not be considered interchangeable “TI markers.” They capture different layers of the TI cascade and have different specificity, stability, and feasibility. For example, H3K4me3/H3K27ac and open-chromatin assays are mechanistically proximal but require standardized chromatin immunoprecipitation or ATAC-based workflows, costly instrumentation, and stringent quality control; they are currently research-grade rather than point-of-care (67, 94). Monocyte cytokine production after LPS re-stimulation is functionally relevant and flow- or ELISA-compatible, but it is influenced by disease activity, corticosteroids, biologics, infection, and sample processing time (59–61). Fecal MPO and serum IL-17/IL-22/UII reflect mucosal or systemic inflammation rather than innate immune memory per se; they may therefore predict cardiovascular risk through general inflammatory burden rather than TI-specific reprogramming (48, 95–97). A recent translational review highlighted that although increasing experimental and clinical evidence supports a role for TI in sustaining chronic intestinal inflammation, robust clinical validation in human IBD cohorts is currently lacking and the role of TI in IBD remains largely confined to early preclinical evidence (98). Therefore, a clinically useful TI biomarker panel should include at least one functional marker (e.g., monocyte TNF-α/IL-6 after LPS), one epigenetic marker (e.g., H3K4me3/H3K27ac at TNF/IL6 promoters), and one metabolic marker (e.g., lactate or α-ketoglutarate), with pre-specified assay standardization and reference ranges (67, 94). At present, evidence supports analytical validity for some markers, but not clinical validity or clinical utility. Validation must be prospective, multicenter, and incremental: markers should improve discrimination or reclassification beyond Framingham/SCORE2 and hs-CRP, with reporting of NRI/IDI, calibration, and cost-effectiveness (99, 100). Until such data are available, TI markers should be regarded as promising research tools, not routine clinical tests.

## 8 Therapeutic strategies targeting TI in IBD-associated CVD: a critical appraisal

Existing evidence for TI-targeting therapies in IBD-associated CVD is mostly preclinical, based on surrogate endpoints, or extrapolated from other inflammatory diseases. The following appraisal therefore prioritizes IBD-specific feasibility, safety, and trial readiness rather than listing agents. No TI-targeting therapy has yet demonstrated cardiovascular benefit in IBD, and the field remains at a hypothesis-generating rather than therapeutic development stage.

### 8.1 Anti-inflammatory and biologic therapies: the most immediate but still unproven route

Among TI-targeting therapies, TNF-α inhibitors (e.g., infliximab) are the most clinically advanced candidates: they can partially reverse monocyte TI and reduce IL-1β/IL-6 production, and IBD patients already receive them for intestinal disease (31). However, it remains unproven whether this reverses HSC epigenetic memory or reduces hard cardiovascular endpoints; prospective trials should therefore embed cardiovascular imaging (carotid intima-media thickness, coronary calcium, flow velocity reserve) and TI assays into existing IBD biologic cohorts (32, 78–80). IL-12/23 inhibitors (e.g., ustekinumab) may attenuate TI memory by blocking IL-23 signaling, but cardiovascular outcome data are lacking (31). Glucocorticoids rapidly suppress inflammation but worsen metabolic risk and should not be repurposed for TI modulation beyond acute disease control (31). Thus, anti-inflammatory drugs are attractive but should not be claimed to be cardiovascular protective without endpoint-driven evidence.

### 8.2 Epigenetic modifiers: mechanistically rational but limited by specificity and safety

HDAC inhibitors, DOT1L inhibitors, and DNA methyltransferase inhibitors can reverse pro-inflammatory epigenetic marks in preclinical models, but they face major barriers in IBD-associated CVD: poor cell-type specificity, risk of broad transcriptional effects, potential infection/cancer concerns, and lack of cardiovascular endpoint data (2, 44, 101). Clinical trials in IBD have evaluated histone-modifying enzyme inhibitors such as ITF2357 in moderate-to-severe Crohn’s disease, but these trials focused on mucosal healing rather than cardiovascular outcomes, and systemic safety concerns remain unresolved (101). Notably, in mice with myeloid-specific Dot1l deficiency, DOT1L loss paradoxically promotes macrophage hyperactivation and reduces atherosclerotic plaque stability, indicating that DOT1L promotes atherosclerotic plaque stability in this context and that DOT1L inhibition may be harmful. This further argues against DOT1L inhibition as a therapeutic strategy (102). Their clinical development should focus on monocyte- or HSPC-targeted delivery, short exposure, and biomarker-guided dose selection rather than systemic chronic administration (67). However, given the absence of cardiovascular endpoint data and unresolved safety concerns, these agents are not ready for clinical translation in IBD-associated CVD.

### 8.3 Metabolic interventions: repurposing opportunities with realistic translational potential

Three classes of metabolic agents target the metabolic foundation of TI: glycolysis inhibitors (2-DG), glutaminase inhibitors (DON), and mevalonate-pathway inhibitors (statins) (31, 44). Among these, statins are the most feasible because they are already used in cardiovascular prevention and can prevent β-glucan- and oxLDL-induced TI *in vitro* by inhibiting the mevalonate pathway (46). However, a prospective cohort study in patients with familial hypercholesterolemia showed that statin treatment does not reverse the trained immune phenotype of circulating monocytes despite normalization of cholesterol levels, suggesting that TI established by chronic endogenous stimuli may be resistant to mevalonate-pathway inhibition once fully reprogrammed (103). This finding has direct implications for IBD: statins may prevent *de novo* TI induction but may not reverse pre-existing TI memory in patients with longstanding disease. 2-DG and DON are not clinically mature; 2-DG causes extensive, non-specific suppression of granulocyte biofunctions, such as cell-to-cell signaling and nucleic acid metabolism, indicating potential unexpected consequences (104). Metabolic interventions should therefore be tested as adjuncts, with TI biomarkers as mechanistic readouts and cardiovascular imaging or events as outcomes. At present, none has demonstrated cardiovascular benefit in IBD.

### 8.4 Microbiome modulation: biologically compelling but heterogeneous and underpowered

Probiotics, FMT, and selective antibiotics may reduce microbial translocation and TI induction, but current evidence is heterogeneous, donor- and disease-subtype-dependent, and almost entirely based on inflammatory surrogates rather than cardiovascular outcomes (12, 38, 47, 75). In mouse models of chronic colitis, therapeutic FMT rescued heart function and mitigated fibrosis in both DSS-treated and *Il10*−/− models, providing the first causal evidence that correcting gut dysbiosis can reverse colitis-induced cardiac remodeling (105). However, *Il10*−/− mice demonstrated relatively less cardiac recovery following FMT, highlighting IL-10’s cardioprotective and anti-inflammatory contribution and indicating that host genetic and immunological context modulates the cardiovascular response to microbiome intervention (105). FMT should not be recommended for cardiovascular risk reduction in IBD outside clinical trials. Precision microbiome strategies should be stratified by CD versus UC, dysbiosis pattern, and TI phenotype, with strict safety monitoring (31, 38).

Overall, the field needs a tiered therapeutic framework: (i) repurpose existing IBD biologics and statins with embedded cardiovascular/TI endpoints; (ii) develop targeted epigenetic or metabolic modulators only with strong safety and delivery solutions; and (iii) reserve microbiome interventions for rigorously controlled trials. Without this critical appraisal, a simple list of agents may overstate clinical readiness (**Table 4**).

**Table 4:** Therapeutic strategies targeting TI in IBD.

Category	Agent	Rationale	Evidence in IBD	References
Anti-inflammatory	TNF-α inhibitors (infliximab)	Reverse trained monocyte phenotype; reduce pro-inflammatory output	Partially effective; no CV endpoint data	(31)
Epigenetic modifier	HDAC inhibitors, DOT1L inhibitors	Reverse pro-inflammatory histone marks	Preclinical efficacy in IBD models; DOT1L inhibition may be harmful	(2, 44, 67)
Metabolic	2-Deoxy-D-glucose, statins	Block metabolic reprogramming; inhibit mevalonate pathway	Statins prevent *de novo* TI *in vitro*; no CV endpoint data	(31, 32, 44, 106)
Microbiome	Probiotics, FMT	Reduce microbial translocation	Heterogeneous; no CV endpoint data	(12, 38, 75)

## 9 Future directions and clinical translation challenges

### 9.1 Mechanistic insights: single-cell and spatial omics

How IBD-specific microbial products induce TI via pattern recognition receptors is increasingly clear. Dysbiosis releases molecular patterns such as LPS, which trigger epigenetic reprogramming and metabolic remodeling, establishing a persistent pro-inflammatory state (75). CD and UC differ in TI features (CD via NOD2, UC via TLR4/TLR9), and clinical studies confirm that patients with CD have a higher risk of stroke (98). However, whether single-cell and spatial omics can identify distinct “trained” monocyte/macrophage subsets in IBD-associated CVD remains an open question. Future studies should determine whether such subsets show sustained H3K4me3/H3K27ac alterations, whether their cytokine production correlates with arterial stiffness, and whether their spatial distribution in plaque or myocardium differs between CD and UC. These advances will be crucial for discovering new therapeutic targets.

### 9.2 Biomarker validation: multicenter prospective cohorts

Clinical validation of TI markers requires a multipronged approach. Prospective cohort studies of serum markers such as UII, IL-17, and IL-22 are needed to assess their predictive value independent of conventional risk factors (107, 108). Assay methods must be standardized: fecal MPO activity, serum elafin and others can be reliably measured by ELISA (97, 109), and international multicenter comparisons should be promoted. Integrating TI markers into traditional risk scores (e.g., the Framingham score) could improve predictive accuracy; multi-marker combined models have already shown high diagnostic performance (110, 111). The net reclassification improvement index and similar metrics should be used to quantify their incremental value. Thus, establishing a rigorously validated, multidimensional panel of markers is essential for translating TI into clinical practice.

### 9.3 Intervention studies: multi-strategy clinical trials

Clinical trials targeting TI are essential for translation. Existing anti-inflammatory drugs such as anti-TNF-α should be prospectively evaluated for their modulation of TI, with trial designs including long-term follow-up and subclinical atherosclerosis markers (112). Novel epigenetic modifiers and metabolic inhibitors require rigorous safety assessment to avoid compromising immune defense or disrupting metabolism (113, 114); early-phase trials should include dose escalation and data safety monitoring. RCTs of microbiome modulation strategies (probiotics, FMT) need strict placebo controls, co-primary endpoints of cardiovascular events and inflammatory markers, and consideration of IBD subtype differences (115). In summary, clinical trial designs for multiple strategies—from anti-inflammatory drugs and epigenetic interventions to microbiome modulation—must balance efficacy and safety to maximize cardiovascular benefit.

### 9.4 Personalized strategies: stratified medicine

Establishing personalized treatment strategies requires integrating multiple layers of information. Patients with IBD exhibit a “lipid paradox,” and traditional risk scores may underestimate true risk (80, 116). Integrating markers such as hs-CRP, zonulin and calprotectin together with GWAS data can build more accurate predictive models (117). Stratification by TI phenotype: high-responder phenotypes should prioritize biologics with cardiovascular protective effects (e.g., anti-TNF-α) (99); low-responder phenotypes should focus on conventional risk factor management (114); corticosteroid use should be reduced, and lifestyle interventions applied to those with metabolic syndrome (116). Ultimately, integrating data from the gut microbiome, epigenome, and metabolome, by identifying high-risk microbial community patterns through fecal metagenomics (100), combined with changes in miRNA profiles and metabolites (short-chain fatty acids, trimethylamine N-oxide) (96), provides a “molecular portrait” of the patient. This portrait guides the selection of optimal anti-inflammatory, microbiome-modulating and cardiovascular protective strategies to achieve precision intervention (13, 118). Together, these integrated strategies can shift the management of IBD-associated cardiovascular risk from a one-size-fits-all approach to a personalized era, ultimately improving long-term patient outcomes (**Figure 2**).

**Figure 2:**
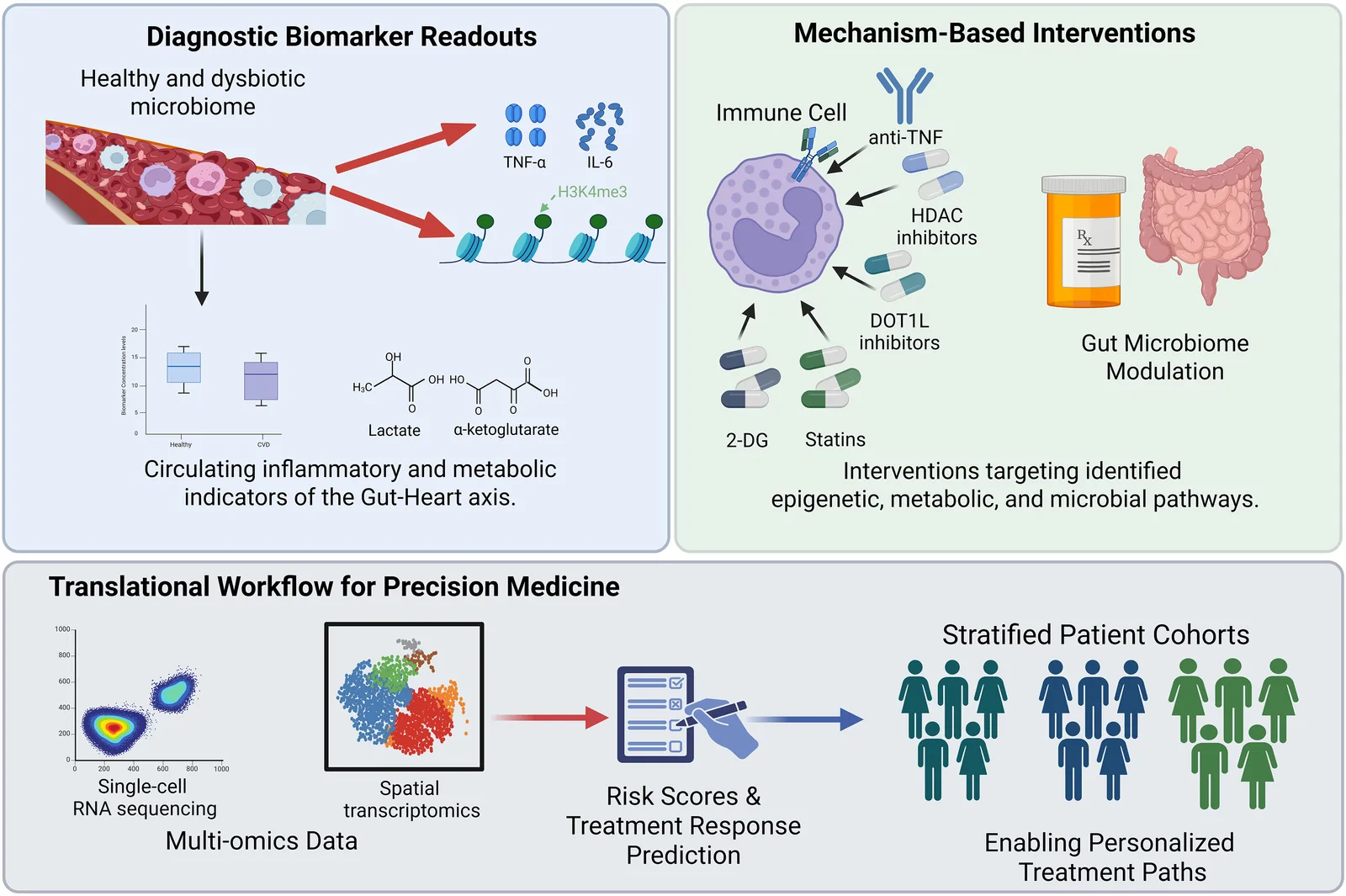
TI in IBD-associated CVD: biomarkers, interventions, and precision medicine.

Diagnostic readouts include functional, epigenetic, and metabolic markers. Mechanism-based interventions target epigenetic, metabolic, and microbial pathways. Multi-omics data integrate with risk scores and treatment response prediction to enable stratified patient cohorts and personalized treatment paths. Arrows indicate workflow direction and mechanistic targeting.

## 10 Limitations

Several limitations should be acknowledged. IBD is heterogeneous in phenotype, activity, duration, and treatment history, all of which may differentially affect TI induction and assessment (31, 48). Immunomodulatory therapies commonly used in IBD, including anti-TNF agents and glucocorticoids, can modulate innate immune function and epigenetic programming, potentially confounding TI measurements in observational studies (31). Distinguishing TI from general inflammatory burden is difficult because many TI markers overlap with conventional inflammatory readouts (10, 48). Standardized assays for TI markers are lacking, with substantial variability in stimulation protocols, timing, and readouts (10). Reverse causation cannot be excluded, as cardiovascular disease and its risk factors may themselves promote intestinal inflammation and microbial translocation (25). Prospective longitudinal studies with standardized assays and careful confounder adjustment are needed.

## 11 Conclusion

The epidemiological association between IBD and CVD is well established, yet conventional risk factors do not fully explain it. The concept of TI offers a new perspective on the immunological basis of increased cardiovascular risk in patients with IBD. Current evidence indicates that IBD-related gut dysbiosis and systemic inflammation induce a persistent hyperresponsive state in circulating monocytes and bone marrow hematopoietic stem cells through epigenetic reprogramming and metabolic remodeling. This TI state may contribute to an exaggerated inflammatory response to atherogenic stimuli, thereby accelerating plaque progression, increasing plaque vulnerability, and contributing to myocardial injury and heart failure.

This mechanistic framework has important translational implications. First, markers of TI, such as monocyte cytokine production capacity and histone modification levels, could serve as novel biomarkers for cardiovascular risk in IBD patients, complementing traditional risk stratification tools. Second, therapeutic strategies that target TI, including anti-inflammatory drugs, epigenetic modifiers, metabolic interventions and microbiome modulation, show promise for improving cardiovascular outcomes in IBD.

However, the evidence must be interpreted with caution. Most current data come from animal models and *in vitro* studies, and direct causal evidence in human IBD cohorts is still lacking. The mechanistic link between IBD-associated TI and cardiovascular outcomes should therefore be regarded as a hypothesis supported by converging preclinical and translational evidence rather than an established causal pathway. Clinical translation faces several challenges: standardizing methods for dynamic monitoring of TI status, ensuring the safety of long-term interventions, and developing personalized treatment strategies. Future research should focus on large-scale prospective cohorts to validate the clinical value of TI markers, dissect the molecular mechanisms of the gut–immune–cardiovascular axis, and evaluate the cardiovascular benefits and risks of TI-targeting strategies through randomized controlled trials. Only by integrating basic mechanistic research with clinical translation can the ultimate goal of improving cardiovascular health and quality of life in IBD patients be achieved.

## Glossary

2 DG: 2 Deoxy D glucose
DON: 6 Diazo 5 oxo L norleucine
AIEC: Adherent invasive Escherichia coli
BCG: Bacille Calmette Guérin
CRP: C reactive protein
CVD: Cardiovascular disease
CD: Crohn's disease
FMT: Faecal microbiota transplantation
GMPs: Granulocyte monocyte progenitors
HSCs: Haematopoietic stem cells
HF: Heart failure
HFpEF: Heart failure with preserved ejection fraction
HSPCs: Hematopoietic stem/progenitor cells
HDL: High density lipoprotein
HDACs: Histone deacetylases
IBD: Inflammatory bowel disease
IRI: Ischemia/reperfusion injury
IHD: Ischemic heart disease
LDL: Low density lipoprotein
MACE: Major adverse cardiovascular events
MMPs: Matrix metalloproteinases
MSU: Monosodium urate
MPO: Myeloperoxidase
MI: Myocardial infarction
oxLDL: Oxidized low density lipoprotein
PAMPs: Pathogen associated molecular patterns
ROS: Reactive oxygen species
SCFA: Short chain fatty acid
TI: Trained immunity
UC: Ulcerative colitis
UII: Urotensin II
VEGF: Vascular endothelial growth factor
VTE: Venous thromboembolism
